# An Implementation of Trust Chain Framework with Hierarchical Content Identifier Mechanism by Using Blockchain Technology

**DOI:** 10.3390/s22134831

**Published:** 2022-06-26

**Authors:** Hsing-Chung Chen, Bambang Irawan, Pei-Yu Hsu, Jhih-Sheng Su, Chun-Wei (Jerry) Lin, Prayitno, Karisma Trinanda Putra, Cahya Damarjati, Chien-Erh Weng, Yao-Hsien Liang, Pi-Hsien Chang

**Affiliations:** 1 Department of Computer Science and Information Engineering, Asia University, Taichung 413305, Taiwan; bambang.irawan@esaunggul.ac.id (B.I.); hcliebe2019@gmail.com (P.-Y.H.); 108121019@live.asia.edu.tw (J.-S.S.); prayitno@polines.ac.id (P.); 108021072@live.asia.edu.tw (Y.-H.L.); 2 Department of Medical Research, China Medical University Hospital, China Medical University, Taichung 404327, Taiwan; 3 Department of Computer Science, Esa Unggul University, West Jakarta 11510, Indonesia; 4 Department of Computer Science, Electrical Engineering and Mathematical Sciences, Western Norway University of Applied, 5063 Bergen, Norway; jerrylin@ieee.org; 5 Department of Electrical Engineering, Politeknik Negeri Semarang, Semarang 50194, Indonesia; 6 Department of Electrical Engineering, Universitas Muhammadiyah Yogyakarta, Bantul 55183, Indonesia; karisma@ft.umy.ac.id; 7 Department of Information Technology, Universitas Muhammadiyah Yogyakarta, Bantul 55183, Indonesia; cahya.damarjati@umy.ac.id; 8 Department of Telecommunication Engineering, National Kaohsiung University of Science and Technology, Kaohsiung 81157, Taiwan; 9 Information Management Center, Taichung City Government, Taichung 407610, Taiwan; ps491@taichung.gov.tw

**Keywords:** trust chain framework, blockchain, comprehensive identification mechanism, manufacturing innovation and product innovation

## Abstract

Advances in information technology (IT) and operation technology (OT) accelerate the development of manufacturing systems (MS) consisting of integrated circuits (ICs), modules, and systems, toward Industry 4.0. However, the existing MS does not support comprehensive identity forensics for the whole system, limiting its ability to adapt to equipment authentication difficulties. Furthermore, the development of trust imposed during their crosswise collaborations with suppliers and other manufacturers in the supply chain is poorly maintained. In this paper, a trust chain framework with a comprehensive identification mechanism is implemented for the designed MS system, which is based and created on the private blockchain in conjunction with decentralized database systems to boost the flexibility, traceability, and identification of the IC-module-system. Practical implementations are developed using a functional prototype. First, the decentralized application (DApp) and the smart contracts are proposed for constructing the new trust chain under the proposed comprehensive identification mechanism by using blockchain technology. In addition, the blockchain addresses of IC, module, and system are automatically registered to InterPlanetary File System (IPFS), individually. In addition, their corresponding hierarchical CID (content identifier) values are organized by using Merkle DAG (Directed Acyclic Graph), which is employed via the hierarchical content identifier mechanism (HCIDM) proposed in this paper. Based on insights obtained from this analysis, the trust chain based on HCIDM can be applied to any MS system, for example, this trust chain could be used to prevent the counterfeit modules and ICs employed in the monitoring system of a semiconductor factory environment. The evaluation results show that the proposed scheme could work in practice under the much lower costs, compared to the public blockchain, with a total cost of 0.002094 Ether. Finally, this research is developed an innovation trust chain mechanism that could be provided the system-level security for any MS toward Industrial 4.0 in order to meet the requirements of both manufacturing innovation and product innovation in Sustainable Development Goals (SDGs).

## 1. Introduction

In the last decade, the manufacturing system (MS) has been one of the major concerns in the development of Industry 4.0. Both manufacturing and assembly are competitive sectors, which is the target for parties who take illegal profits. Many interrelated parties take roles in this sector. Manufacturing industries include around 628,536 companies in the US and contribute to 24% of the US GDP according to statistics in 2020 [1]. Moreover, geolocation also has implications; for instance, although the designs of the components are still dominated by the US, Taiwan and South Korea dominate the chip-manufacturing industry, contributing to 83% of global production [2]. Then, in late 2020, a chip shortage emerged by the industry in the downstream sector, including the car and computer industries [3]. Manufacturing enterprises must shift towards MS to maintain advantages in a competitive global market [4,5].

Mass customized zero-defect production is a significant challenge in MS [6,7]. The more massive the demand for customized products, the higher the potential for counterfeiting. Furthermore, some counterfeiters have begun manufacturing their products in the same factory as the original product [8]. To solve the above challenges, manufacturing enterprises need to maintain their innovation. For instance, to increase production efficiency and save manufacturing costs, the company needs to increase production capacity using some of the latest technological developments, such as the Industrial Internet of Things (IIoT) [9]. Next, manufacturing companies need to collaborate with several suppliers and other manufacturing industries to produce final collaborative products that meet global market requirements. Finally, transparency during the production phases needs to be improved, providing consumers with product safety and well-tracked warranty claims. To adopt cutting-edge, manufacturers must implement advanced information technology. Unfortunately, the MS foundation is based on centralized computing with poor flexibility [10]. In addition, the development of trust imposed during crosswise collaborations with suppliers and other manufacturers is poorly maintained, thus creating complex, poorly managed, and error-prone collaborations [11]. Therefore, trustable methods and chains are the only way to track product originality and reduce counterfeits.

As one of the advances in new-generation information technologies, blockchain improves digital transactions in efficiency, transparency, and security. As a new paradigm, the comprehensive identification mechanism using blockchain technology states that data could be communicated, tracked, and stored securely [12]. The trust chain enables distributed trust to be scalable. An immutable record of the transaction is created by allowing two parties to agree on a trade or transaction and storing the proof of the transaction in a firm form. A blockchain consists of a series of blocks that record each transaction. Every peer maintains a copy of their blockchain, containing records of all transactions. Blockchain changes the paradigm of a conventional centralized system into a decentralized trusted system. The existing centralized architecture suffers from the weakness as follows. (1) IIoT is built because all end nodes, that is, connected to a centralized system, are initially verified by the system. After one of the end nodes is disconnected from the system, there is no guarantee that the system could trust the same node. A re-validation process needs to be performed by the system, and the end node is broken in the system. (2) The performance of the massive centralized systems is greatly affected by latencies and errors. Moreover, if new nodes repeatedly manage further sub-branches inside the old one, a longer network chain will be developed, thus increasing the latencies and errors. (3) The competitive manufacturing sector is forcing companies to switch to current MS because customers now want to be involved in the manufacturing process to make a more customized and personalized product. Meanwhile, current MS faces security problems because the system does not guarantee the validity of new users. Blockchain technology combined with a decentralized database offers the practical concept of realizing decentralized current MS. However, in the industrial sector, blockchain technology is only a concept and lacks implementation for the current MS [13,14,15]. It is still challenging to provide a consensus technique suitable for applications [16] in the industrial domain with reliable validation techniques.

The main contributions of this paper are stated below.

(1) Implement the consortium blockchain via applying the Quorum blockchain network (QBN). Combining it with a decentralized database, the InterPlanetary File System (IPFS) provides flexibility, traceability, and security mechanisms for current MS.
(2) Both nature Merkle trees in QBN plus Merkle DAG (Directed Acyclic Graph) are employed and implemented for storing the CIDs and the corresponding product version files of ICs, modules, and systems, individually.
(3) The web-based DApp is implemented by using the smart contracts designed in this study to interact with the QBN together with the IPFS collaboration framework, where the hierarchical content identifier mechanism (HCIDM) is first proposed and designed for each customized system product in order to create the system-level trust chain and easily validate or trace the trusted product versions of IC, module, and the customized system itself, comprehensively.
(4) The consensus-oriented transaction logic based on the voting-based consensus protocol [16] in the implemented QBN shows that it could improve latency and throughput compared with the previous consensus protocols [17].

In addition, the prototype concept’s implementation is presented to meet the requirements of the MS plus consortium blockchain for the manufacturing industry. The evaluation will be demonstrated that the proposed idea is practical and efficient with a flexible, traceable, and secure decentralized working environment in this paper.

Finally, the remains of this paper are organized below. Section 2 discusses related works, while in Section 3, we present the system model used in this study. Section 4 evaluates experimental results. Section 5 presents discussions and analyses. Finally, Section 6 summarizes this study.

## 2. Related Works

In this section, the current MS and its relative technologies will be introduced. Next, some solutions for implementing different MSs using the blockchain technology approach will be discussed. Additionally, the smart contracts will be also explained. The distributed data storage system, for example, IPFS will be addressed. Finally, Dapp will be given short descriptions and informed on how to co-work with smart contracts.

Currently, MS is still improving industrial technology for satisfying the requirements of manufacturing efficiency, transparency, and security [12]. In the other words, MS still pays his full efforts to optimize production and product transactions as a broad manufacturing concept toward Industry 4.0 by integrating advanced manufacturing with information engineering [18]. With advancements in information engineering, these cutting-edge models might likely be employed to address the shortcomings of the present production model [19,20,21]. Furthermore, with the emergence of the IIoT concept based on small intelligent sensors, the future production line is supported by heterogeneous sensory modules that functionally collaborate to support the manufacturing process [22]. With more functional nodes connected in a centralized IIoT system, manufacturing enterprises must shift towards an MS with many promised benefits, for example, intelligent, and remote management. Each functional module is fitted with numerous sensory units that monitor the module’s components to ensure a more precise perception and grasp of the manufacturing process. Thus, management parties could precisely monitor each component, improving their oversight of complex parts to ensure that production phases go smoothly. However, the MS concept needs to be revised with a new approach with increasingly varied product demands. The MS should bring customers closer to the manufacturing process, making it easier to get personalized custom products [12,23]. However, this centralized private system, for example, the current MS, cannot handle various problems, including counterfeiting [24], copyright [25], and consumer protection [26], due to the lack of united records from all parties who participate in production and distribution processes. Even though the manufacturer is an enterprise-level party, in the process, they cooperate with third parties in a multilevel subcontracting mechanism making each base component hard to be traced. Therefore, a decentralized, simple, traceable, and secure network is required in the development of current MS, and this is what blockchain technology offers.

Blockchain defines that although they support both private and public networks, the data can be confidently communicated, traced, and securely stored [27]. In a concept that combines MS and IIoT, several functional sensors measure real-time data from the production lines [18]. This intelligent concept paves the way for better monitoring and understanding of industrial phases to perform effective and efficient production. Moreover, the current MS concept that brings consumers closer to the manufacturing processes could expose the system to the outside world, thus increasing the chance of cyberattacks [28]. Traditional protection mechanisms fail to protect centralized systems due to the low computing resource at edge devices [29]. The deployment of centralized systems might be more expensive for companies as powerful central servers and regular maintenance are required. Table 1 presents various solutions for implementing different MSs using the blockchain technology approach. 

In general, blockchain could be constructed with four parts consisting of the ledger, smart contracts, consensus, and cryptography [30]. In a blockchain network, the ledger archives all transactions by the parties, and each party keeps a copy of it. A smart contract authorizes access to the ledger. A consensus is used to synchronize the ledger across the network. Finally, the cryptography mechanism envelops the network transactions and the ledger data, making these binds hard to break and trace by eavesdroppers. In terms of blockchain platforms, there are two kinds of platforms, namely, without authorization (i.e., permissionless blockchain) and with authorization (i.e., permission blockchain) [31]. Any party could participate in the permissionless blockchain network without authorization, e.g., Bitcoin [32] and Ethereum [33].

On the contrary, the permission blockchain implies that members should have a specific identity, that is, Hyper Fabric [34] and Quorum [35]. Substantially, both higher throughput and lower latency are performed by permission blockchain due to their nature of consensus. One consensus algorithm in a permissionless blockchain, such as Proof of Works (PoWs), is complex and costly to handle Sybil attacks [36]. On the other hand, PoA consensus in a permissioned blockchain ensures that all members are equally motivated to succeeding their network, although with a lower number of members but with a more trusted identity [37]. This concept is more implementable for enterprise-level applications such as manufacturing industry cases. 

In addition, the ledger functions in a networked environment based on decentralization in the blockchain world. Each ledger block contains data regarding platform transactions. The block is produced using the data, hash function, and hash value from the preceding block. The process is called a chain [38,39]. According to most definitions, a blockchain is a decentralized database where cryptographic signatures verify transactions. Consensus algorithms like proof of stake (PoS) and PoW allow blockchain to confirm transactions based on the agreement of all peers [40,41,42]. Both PoS and PoW protocols ensure that transactions between peer nodes in a blockchain network are safe and dependable. Public, private, or consortium are the classifications in blockchain [4]. The public blockchain network is an architecture that could read given notes. It denotes being accessible to anyone on Earth, validating its status as a node, and participating in consensus. Crypto-economics is primarily concerned with the convergence of economic incentives with cryptographic verification via proof-of-work (Bitcoin) or proof-of-stake (Ethereum). Blockchain technology is generally regarded as fully decentralized. One downside is the substantial computational power (resources) necessary to maintain large-scale distributed ledgers, crucial in densely deployed IoT networks. By implementing rigorous constraints, the shared blockchain network protects end-users from developers. A fully private blockchain gathers written permissions into the hands of a single institution. The scope of reading permissions could be public or arbitrary. In many circumstances, internal applications such as database management and auditing should not require general readability, while public audibility may be desired in others. A consortium blockchain is a multi-center blockchain system built and maintained collaboratively by an agreed group. Typically, each node corresponds to an entity organization. After finishing both authentication and authorization processes, each node can join, access, and transmit transactions. In addition, each consortium member has specific data permissions. The blockchain consortium is the dominant approach as it enables voluminous and robust collaboration across companies and organizations, promoting the healthy and orderly development of the blockchain industry. 

Next, smart contracts are computer programs that facilitate, verify, and enforce legal transactions. Implemented through blockchain transactions, linked to cryptocurrencies, and an input interface for contracting parties. When implemented on the blockchain, smart contracts become autonomous entities capable of performing specific tasks automatically when certain conditions are met. The execution of smart contracts on the blockchain is not subject to censorship, downtime, fraud, or third-party interference. Ethereum is the most widely used smart contract platform in the sector. Smart contracts provide distributed trusted computing on a blockchain platform. Embedded hardware and software translate the current contract clauses into smart contracts to verify that the contract is eligible. Code-based smart contracts can interact with other contracts, make decisions, store data, and send tokens/money to other contracts.

Moreover, IPFS is a distributed data storage system that addresses content and assigns a unique hash to each item stored [43]. IPFS CID version “0” and “1” enables a high-throughput, resource-efficient storage paradigm with concurrent access. IPFS generates a hash that is 46 bytes in length as a result, putting transaction data in IPFS, and the hash generated by IPFS in the blockchain block results in significant savings in storage space [43]. A larger file is fragmented and stored on multiple nodes. The term “node” refers to the various computing devices that comprise the IPFS network. When data are retrieved, it is associated with a hash key based on the content to which it refers. Using these hash keys, one could simultaneously access data from several nodes. The term “content-addressable storage” refers to this principle.

Additionally, a decentralized application (DApp) [44] is a decentralized network-based program that combines smart contracts and a user interface on the front end due to the open and transparent nature of Ethereum smart contracts, which are analogous to an open API [40]. Our DApp could integrate smart contracts established by others. The backend code of DApp is hosted on a decentralized peer-to-peer network. Compared to legacy systems, which rely on a centralized server to execute backend code. In general, a programmer could easily write a DApp frontend code and user interface in any language to make calls to its backend (such as an App). Additionally, it is possible to host its frontend on a distributed storage system such as IPFS [45].

The primary purpose of distributed data storages for blockchain is to provide storage for all transaction records. It does not provide a huge distribution data set for all data types. Blockchain is currently the most efficient and secure methods [46,47,48] of storing information as a distributed ledger, facilitating data sharing among multiple parties under their collaborations. Moreover, there are many blockchain applications [49,50,51,52,53] by using and deploying blockchain technologies to provide the security in distribution system. IPFS is a peer-to-peer decentralized file system [45]. Its protocol is used to create a network infrastructure similar to the Bitcoin blockchain protocol, with the advantages of storing immutable data and removing redundant files on the network. In other words, IPFS provides a high-throughput, content-addressed block storage model, and content-related hyperlinks in the created network infrastructure. It could form a generalized Merkle DAG [54]. IPFS does also combine decentralized hash tables, incentivized block exchanges, and a self-certifying namespace [55]. IPFS has no single point of failure and nodes do not need to trust each other [55]. Decentralized content delivery saves bandwidth and prevents DDoS attacks that HTTP schemes might encounter [56]. Therefore, blockchain and IPFS could collaborate to produce decentralized storage. It could store and distribute various data types without relying on third parties. The approach of combing both blockchain and IPFS is used to circumvent storage limits and allow secure storage of individual data. It could improve privacy, security, and distributed data management.

The related works, mentioned above, show that the convergences of blockchain and decentralized databases may potentially overcome the drawbacks of the current MS. However, blockchain applications, specifically in the industrial domain, are still in their infancy. Integrating blockchain into a current MS concept is still challenging. Thus, this study shortcoming the above challenges with a practical integration of blockchain technology in current MS scenarios.

In this paper, the consortium blockchain technology, namely the Quorum Blockchain Network (QBN) [45], is used to develop a trust chain system. The quorum blockchain network comprises four critical components:

1. The transaction manager provides access to encrypted transaction data for private transactions, manages locally stored data, and facilitates communication with other transaction managers.
2. A cryptographic enclave is responsible for maintaining private keys and the encryption and decryption of personal transaction data.
3. The Byzantine Fault Tolerance Consensus (BFT) [45] relies on voting participants joining the quorum chain to validate and send it on the network, based on Ethereum capabilities.
4. The network manager manages network access, enabling the creation of a permissioned-network.

## 3. Preliminaries

This section discusses some main concepts underlying the traceability technology of blockchain-based integrated systems, modules, and ICs. To get started, explore the role of blockchain and the IPFS in maintaining immutable records of ownership systems, modules, and ICs and the role of smart contracts in automating the registration, authentication, traceability, and transfer of ownership of systems, modules, and ICs. In this study, the DApp is designed by using a hierarchical blockchain smart contract and interacts with the IPFS collaboration platform.

**Figure 1:**
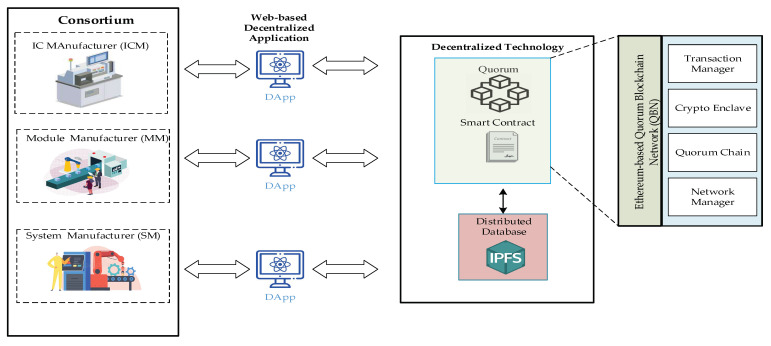
The proposed framework including Quorum blockchain, distributed database (IPFS), and DApp.

In this work, the system manufacturers could use this framework to store necessary information about finished products on blockchain to protect their finished products from copyright infringement and counterfeiting. The Quorum blockchain technology allows encrypted data to be visible only to the parties participating in the transaction, and therefore the system is based on this principle. The production process of a new system starts from the system manufacturer’s request to the module manufacturer. Figure 1 shows the distributed chain flow for system manufacturers, including System Manufacturer (SM), Module Manufacturer (MM), and IC Manufacturer (ICM), forming a chain of alliances. The proposed framework allows the verification and recording of completed data immediately.

Each participant will also register in the proposed blockchain and then be assigned a private-public key pair to identify unique members on the quorum blockchain. The 256-bit private key generates the public key via Elliptical Curve Cryptography [38,46]. The private key must remain secret. Next, every transaction requires a digital signature calculated by Equation (1) below.

(1) $$Kpri{\left[0,1\right]}^{256}.$$

Afterward, a 512-bit public key is produced using the ECDSA (Elliptical Curve Digital Signing Technique) algorithm from the private key. The primary advantage of ECDSA [38,46] over other Public Key Cryptography (PKC) algorithms, such as RSA, is that it needs significantly more minor keys and signatures to obtain a comparable level of security. Signatures are validated using the public key. According to the ECDSA protocol [38,46], Equation (2) is given below.

(2) Kpub=ECDSA(Kpri).

A transaction’s owner embeds their public key and then signs it with the private key. Due to the small size of 512-bit public keys, they could be shared and used in blockchain transactions. To protect against length extension attacks, use a double hash approach in which the 512-bit public key is hashed using SHA-256 and then hashed again with RIPEMD-160 to obtain the 160-bit address by Equation (3).

(3) $$Kaddr=RIPEMD_{160}\left(SHA_{256}\;\left(K_{pub}\right)\right).$$

QBN is critical in authenticating participants and maintaining a directory that associates related party identifiers with transaction addresses. Any identity management system based on blockchain technology can ensure the veracity of user entity information. Users can manage private keys and addresses through digital wallets, conducting any transaction. Digital wallets are a combination of software and hardware or a custom-built hardware device that stores the private-public key and address.

## 4. Our Proposed Framework

In this paper, we propose a framework divided into three phases: Initialization Phase in Section 4.1, Registration Phase in Section 4.2, and Production Phase in Section 4.3.

**Figure 2:**
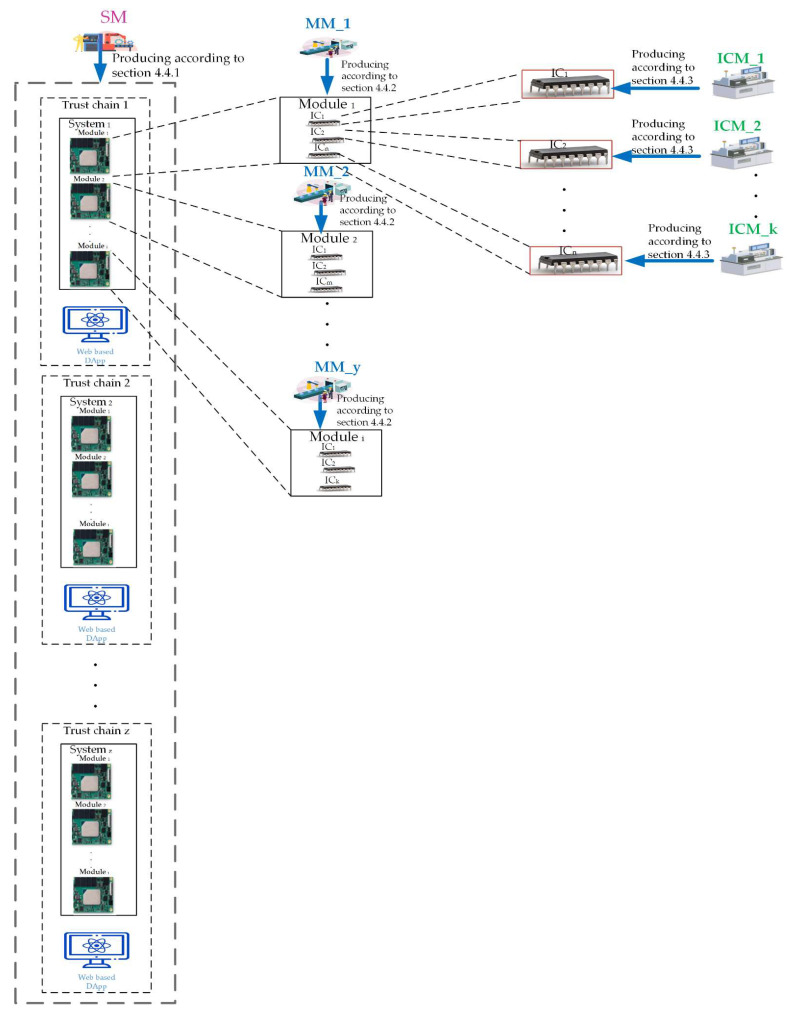
The architecture of this framework.

The architecture of this framework could be illustrated in Figure 2.

### 4.1. Initialization Phase

Each participant with a different role forms a consortium during the initialization phase. Table 2 shows the initial code structure of the smart contracts used in the QBN to organize the trust chain, in which the left-side structure describes a system, modules, and ICs. In contrast, a right-side structure reveals the types of roles involved. In other words, each manufacturer plays a specific role in the consortium. The QBN architecture has a basic chain code structure shown in Table 2, with the symbol notation described in Table 3. When a new system product is created, the left side contains information about the system created by the system manufacturer. In contrast, the right side depicts the structure of the trust chain and the enumeration of the various roles. In addition, the notations are addressed in Table 3.

### 4.2. Registration Phase

**Figure 3:**
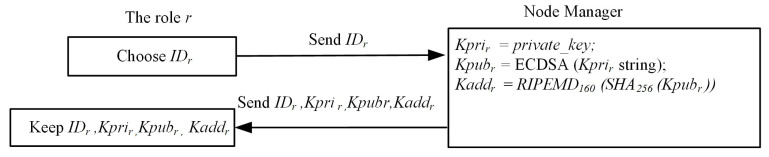
The registration procedure for the participant, e.g., SM, MM, and ICM.

SM, as the node manager, will create the first blockchain network. QBN is a blockchain network that we created. Both MM and ICM join the quorum blockchain network through the SM node manager and get the corresponding public and private keys in QBN. Each role refers to a role node registered in the blockchain system, where the registration procedure is described in Figure 3. In QBN, each participant has a node name, role, IP address, node latency, and an E-wallet application with the account address that is the blockchain address. Moreover, this account address is derived by a public key in the designated blockchain network, where the public key is uniquely associated with a single private key. This public key and private pair are stored in the E-wallet.

The detailed steps for the *Registration Procedure* are described below in this subsection.

***Registration Procedure***

Step 1. The notation $ID_{r}$ refers to all roles (*r* roles) involved in QBN. The corresponding manufacturer then submits his product $ID_{r}$ to the blockchain node, separately, where the blockchain node performs the verification of the legitimacy and then registers the identity to QBN.
Step 2. The node manager will then calculate the public key $Kpub_{r}$ and the private key $Kpri_{r}$ in the blockchain network.
  $$Kpub_{r}=Kpri_{r}G$$
Step 3. After confirming that all participants have been already registered, the node manager will perform Algorithm 1. Each role *r* will then receive the generated $\left(ID_{r},Kpri_{r},Kpub_{r}\right)$ from the node manager.
Step 4. The role *r* in the system receives and saves the signature message parameters.

**Algorithm 1:** Registration to Node Manager.  
***Input***
*:*
$ID_{r}$
*together with its role r;*
  
***Output****:*
$ID_{r},Kpri_{r},Kpub_{r},\;Kaddr_{r}$
*;*
  $Kpri_{r}$ = *private_key; //Node manager will assign a private key to identify*
  *unique member by according to Equtaion (1);*  $Kpub_{r}$
*= ECDSA (*$Kpri_{r}\;$*string); //According to Equtaion (2)*
  $Kaddr_{r}=RIPEMD_{160}\left(SHA_{256}\;\left(Kpub_{r}\right)\right)$*; //According to Equtaion (3)*
  *Function Append (*$ID_{r}$
*string, r stiing,*
$Kpri_{r}$*,*
  $Kpub_{r}$*) {*  *return String*
*“Successful Registration”; // It has been registerd to QBN*
  *}*  ***return*** $ID_{r},Kpri_{r},Kpub_{r}$*,*$Kaddr_{r}$

### 4.3. Production Phase

During the phase of making a new product, the participants involved are SM, MM, and ICM. Algorithms 2 and 3 show the details of these processes for the product design employed in the off-chain. Algorithm 4 is used to create a new system production. These algorithms consisting of Algorithms 2–4 are the main DApps proposed for this framework.

**Algorithm 2:** Ordering Function.

  ***Input**: s, m, ic, Order_Quantity, kaddr_r_*

  ***Output**: String notification*

  ***for** (i: = 0; i < the number of product; i++) {*

   *struct details order {*

   *char   s*
*type [100];*
   *char   m*
*type [100];*
   *char   ic*
*type [100];*
   *Order_Quantity {s [int]; m [int]; ic [int];}*

   *char kaddr_r_ [42]*;
   *}*
  *}*  ***return** String notification*

**Algorithm 3:** Order Verification Function.
 ***Input**:*
*s, m, ic, offering price, Order_Quantity, kaddr_r_*

 ***Output**: String notification, approved price*

  *varchar  offering price;*
  *varchar  approved price;*
  *int*      
*Order_Quantity;*
  ***if** (offering price ==”1”) {*
  *approved price:= offering price*
×
*Order_Quantity;*
  *}*
  ***else** {*
  *result = “Fail”, “Disagreement”;*
  *}*
  ***return** String notification, approved price*


**Algorithm 4:** New System.

  ***Input***
*: ID_SM, ID_MM, S_INF, Signature*

  ***Output**: String notification*

  *load current TP[]*

  ***for** (i:=0; i<S_IDs.length; i++) {*

   *TP[S_ID [ i ]].system_Detail.system_manufacture=ID_SM*
   *TP[S_ID [ i ]].system_Detail.system_information=S_INF*
   *TP[S_ID [ i ]].system_Detail.create_datetime=Datetime.Now()*
   *TP[S_ID [ i ]].system_Detail.module_manufacture=ID_MM*
   *TP[S_ID [ i ]].system_Detail.Signature = Signature*

  *}*

  ***return** String notification*


**Figure 4:**
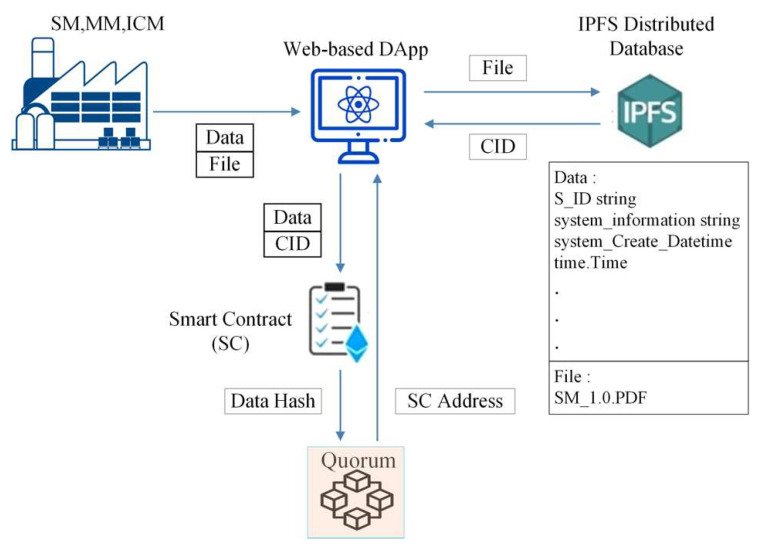
The interaction relationship among DApp, IPFS, and QBN in the proposed framework.

Next, the interaction relationship among DApp, IPFS, and QBN in the proposed framework is illustrated in Figure 4.

### 4.4. The Main Processes for the Implement Platform

**Figure 5:**
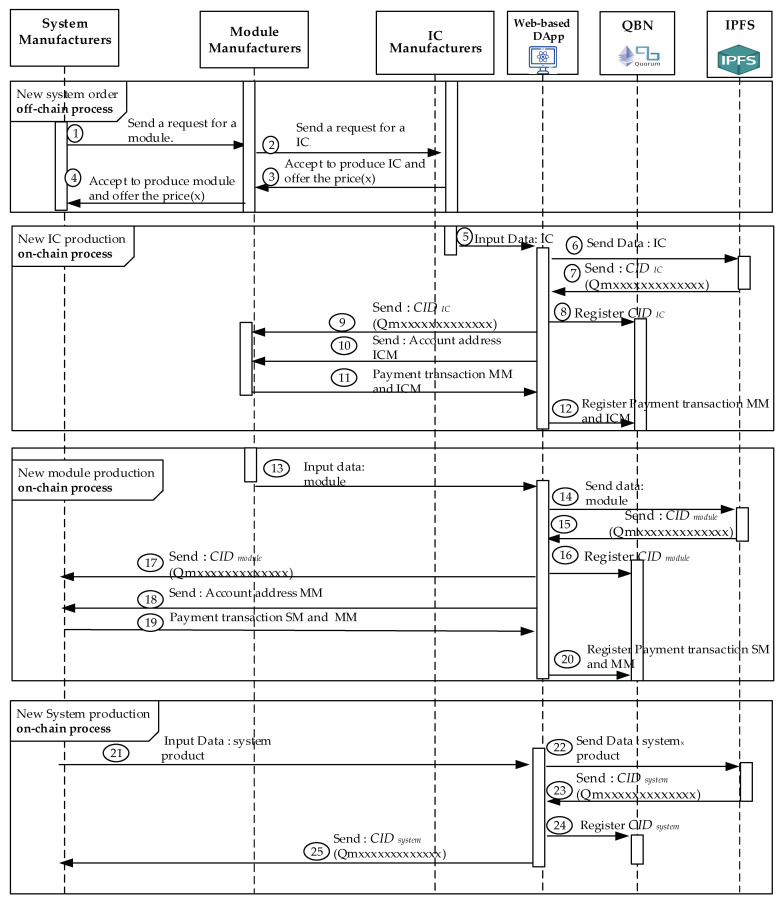
The sequence diagram of stakeholders in producing the system.

After completing the phases of a system design of SM, module design of MM, and IC design of ICM shown in Figure 5 from Step 1 to Step 4, the new IC production, new module production, and new system production are performed from Step 5 to 25, separately. Next, the three main processes for the implementation platform are proposed and presented in this section. The details of the all-party are necessary to realize the three proposed processes. The initial step will register participants in the consortium blockchain [45]. Next, the system, module, and IC will be registered separately in the private Quorum blockchain we created. Both Quorum blockchain and IPFS store every transaction as a transaction record.

IPFS generates the corresponding CID used for authentication throughout a device’s lifetime. It allows traceability and proof of ownership without an explicit need for a trusted intermediary. Furthermore, the authorized parties could utilize the data indexed by CID on the blockchain to authenticate, track, and analyze the product. Figure 5 depicts our proposed approach using the created blockchain.

In the implemented web-based DApp, the CIDs with a hierarchical relationship will be organized by using Merkle DAG via the proposed hierarchical content identifier mechanism (HCIDM) for creating and maintaining the latest trust chain according to both Definition 1 and Definition 2 which are first proposed in this framework. Both Definition 1 and Definition 2 are shown below.

**Definition** **1.** *The latest trust chain is designed via organizing the hierarchical CIDs below.* $\left\{CID_{IC_{1}},CID_{IC_{2}},\ldots ,CID_{IC_{\mu }}\right\}\underline{≺}\left\{CID_{{\mathrm{module}}_{1}},CID_{{\mathrm{module}}_{2}},\ldots ,CID_{{\mathrm{module}}_{\upsilon }}\right\}\underline{≺}\left\{CID_{system_{\chi }}\right\}$ *with the latest version, where the notation “* $\underline{≺}$ *” is the hierarchical organization relationship indicated the trust chain used in this framework.*

**Definition** **2.** *The latest trust chain consisting of the latest version plus the previous versions is also designed via organizing the hierarchical CIDs below. $\begin{array}{l} \left\{CID_{IC_{1}},CID_{IC_{2}},\ldots ,CID_{IC_{\mu }}\right\}\left|\right|\ldots ||\left\{CI{D^{\prime }}_{IC_{1}},CI{D^{\prime }}_{IC_{2}},\ldots ,CI{D^{\prime }}_{IC_{\mu }}\right\}\underline{≺}\\ \left\{CID_{{\mathrm{module}}_{1}},CID_{{\mathrm{module}}_{2}},\ldots ,CID_{{\mathrm{module}}_{\upsilon }}\right\}||\ldots ||\left\{CI{D^{\prime }}_{{\mathrm{module}}_{1}},CI{D^{\prime }}_{{\mathrm{module}}_{2}},\ldots ,CI{D^{\prime }}_{{\mathrm{module}}_{\upsilon }}\right\}\underline{≺}\\ \left\{CID_{system_{\chi }}\right\}||\ldots ||\left\{CI{D^{\prime }}_{system_{\chi }}\right\} \end{array}$ with the latest CIDs version together with the previous CIDs versions, if it is necessary, depending on the rules made by system manufacturer, where the notation “$\underline{≺}$” is also the hierarchical organization relationship.*

In addition, the web-based DApp is designed by using the smart contracts in this study. It could interact with the QBN together with the IPFS collaboration framework, where the HCIDM is first proposed and designed for each customized system product in order to create the system-level trust chain and easily validate or trace the trusted product versions of IC, module, and the customized system itself, comprehensively.

In the real application, each trust chain for each customized system product will be first created by the system manufacturer and stored in the database managed by the owner or buyer for this ‘system product’. Finally. It could be accessed by using the web-based DApp issued by the system manufacturer and managed by the owner or buyer. For instance, the customized ‘System 1’ will be protected via the customized ‘Trust Chain 1’ shown in Figure 2. Next, the trust chain for each customized system product could be employed and co-work with the database managed by the owner’s system administrator.

Therefore, this framework could be employed to trace and validate the current versions or previous versions of ICs, modules, or systems.

#### 4.4.1. New System Producing Process

A blockchain consortium initiates the registration phase of each role in the scheme. QBN authenticates each participant, SM, MM, and ICM. After the authentication procedure is completed, each participant will get the public key, account addresses, and E-node ID sent by the node manager. The participants could communicate through a secured channel. Figure 5 illustrates its detailed procedure. The detailed steps of the production processes are shown in ***New System Producing Procedure*** for the new system.

***New System Producing Procedure***

Step 1. The chain will start when SM wants to create a new system, and SM first submits to MM the production certificate, module order, and production plan.
Step 2. MM will determine the manufacture of the module after receiving the order information from BC. The MM sends a command to the ICM about the required IC information.
Step 3. ICM replies to MM regarding producing the required IC at the agreed price.
Step 4. After receiving information from ICM, MM will provide the response requested by SM via sending the ability to produce the required module and the agreed price. From step 1 to step 4, transaction procedures are carried out off-chain in the order process by SM, MM, and ICM as consortium participants will perform Algorithms 2 and 3.
Step 5. ICM continues to decentralize data to IPFS network nodes after producing new ICs by inputting via DApp.
Step 6. DApp will forward the IC data that has been produced to IPFS to get the *CID_IC_* hash value.
Step 7. Each piece of data is cryptographically hashed to be a secure unique *CID_IC_* by IPFS. Next, it will be then passing it to DApp.
Step 8. The *CID_IC_* obtained will be registered by ICM into QBN through a smart contract.
Step 9. The ICM sends the new *CID_IC_* value for the completed IC to the MM via DApp.
Step 10. The ICM sends account addresses for payment transactions via DApp, where the account addresses are belonging to the account owner, ICM, himself.
Step 11. The MM makes payment transactions to an ICM via DApp.
Step 12. The smart contract we proposed does the transaction processes between ICM and MM regarding IC product, where the payments will be deployed and distributed in QBN.
Step 13. The DApp is used as an interface in order to input the module data. After all material requirements are complete, MM starts the module production. The completed module will be registered to IPFS in order to get the *CID_module_* value for the new module.
Step 14. DApp automatically sends the data module to IPFS and generates the new module’s *CID_module_* value.
Step 15. IPFS sends *CID_module_* data based on the last gathered data for this registered module.
Step 16. After the *CID_module_* value is received, DApp will then automatically register and process it to the created QBN.
Step 17. The new *CID_module_* value of the MM module will be sent to SM via DApp. The MM account address is sent to SM for payment transactions via DApp.
Step 18. SM sends payment transactions to ICM via DApp according to the received account address.
Step 19. The smart contract performs the transactions between MM and SM, which is regarding module payments. Additionally, it will be registered and distributed in QBN.
Step 20. The Smart contract transactions between MM and SM regarding module payments will be registered and distributed in QBN.
Step 21. SM starts to manufacture the new system when all the necessary modules are gathered completely. The new system will be registered its data into IPFS to get the corresponding *CID_System_* value through the DApp.
Step 22. IPFS receives system-generated data sent by DApp, then generates the corresponding *CID_System_* value for the new system and assigns it to SM.
Step 23. IPFS sends back *CID_System_* data to SM.
Step 24. The DApp automatically registers the new system *CID_System_* value consisting of the *CID_module_* values of the assembled module together with the corresponding *CID_IC_* values of IC into QBN. Finally, the CID values will be organized by Merkle DAG [55] and become the hierarchical CID values tree for the trust chain we proposed.
Step 25. Finally, the system data, only stored in IFPS, is registered successfully with the last version via recording its corresponding new *CID_System_* value into the QBN performed by the DApp. Then, the node manager, SM, will perform *Algorithm 4*. The IPFS will deliver the corresponding *CID_System_* value back to SM.

In addition, the DApp, together with the smart contracts, is designed and created by SM for constructing and managing the latest trust chain. 

#### 4.4.2. New Module Producing Process

**Figure 6:**
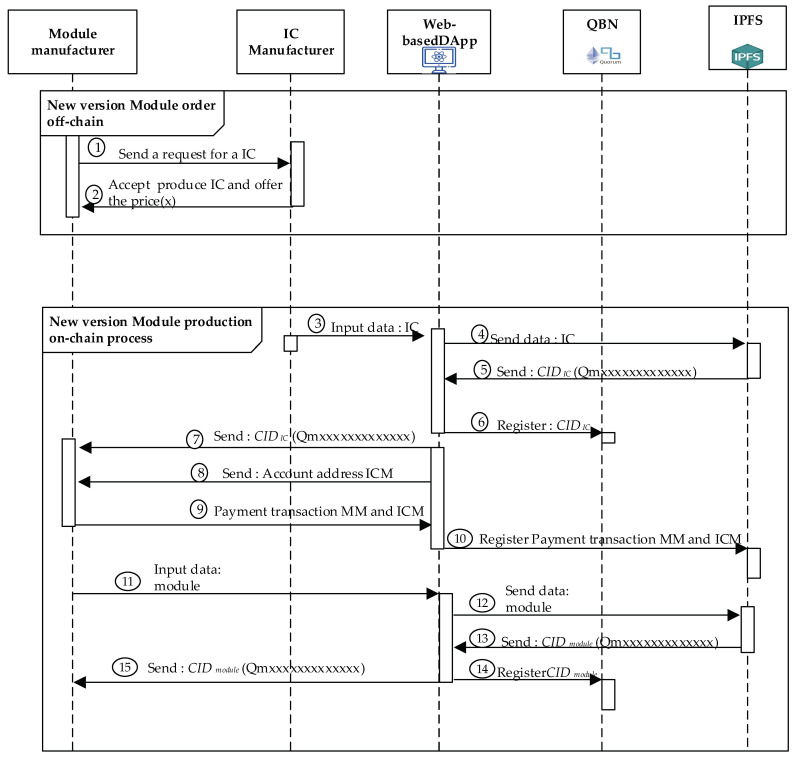
The sequence diagram in producing the new version of module.

MM could manufacture the modules with new versions without requiring an order from SM. Figure 6 shows the sequence in which MM generates a new module version and has the same functionality as the previous module. The new version of the module has additional features, which are the advantages of the original version. The detailed steps of the new module producing processes shown in ***New Module Producing Procedure*** for producing the new version module. The first two steps are used to process the order transactions, which involve the off-chain processed to obtain an MM agreement with ICM. Moreover, Step 3 and the remaining steps of this procedure are the on-chain processes for the QBN we created for these processes. 

***New Module Producing Procedure***

Step 1. The first step starts when MM produces a new version of the module, MM will order the required number of ICs at ICM.
Step 2. After receiving the order of transaction, ICM will check its ability to produce the IC ordered by MM. If ICM can make the IC requested by MM, ICM will provide the price and the ability to produce the IC. Next, both the consortium participants, MM and ICM will perform Algorithms 2 and 3.
Step 3. ICM manufactures ICs according to MM orders of transactions. After production is complete, ICM will input IC data via DApp.
Step 4. DApp will send IC data automatically to IPFS in order to get the *CID_IC_* value.
Step 5. IPFS performs the IC data calculation process in order to generate the *CID_IC_* value and sends it back to DApp.
Step 6. Smart contracts we designed and deployed in QBN will register the *CID_IC_* values via DApp, designed in this project, as intermediaries.
Step 7. DApp sends *CID_IC_* Value back to MM generated by IPFS.
Step 8. DApp sends ICM account addresses to MM for receiving the cryptocurrency during the payment transactions while providing some ICs.
Step 9. MM makes payment transactions to ICM based on account addresses received via DApp.
Step 10. This DApp will register MM payment transactions to ICM by using the smart contract deployed in QBN.
Step 11. After producing the new version of the module, MM will input the new version data module via the DApp.
Step 12. The DApp sends the data entered by the MM to IPFS in order to get the *CID_module_* value for the new version of the module.
Step 13. IPFS generates a new version of the data module and sends its corresponding *CID_module_* value to the DApp.
Step 14. Finally, the module data, only stored in IFPS, is registered with the last version via recording its corresponding new *CID_module_* value into the QBN performed by the DApp.
Step 15. The IPFS will deliver the corresponding *CID_module_* value back to MM.

Moreover, the DApp together with the smart contracts, which are deployed and managed by SM, are performed in order to involve the information of the new module in the new trust chain.

#### 4.4.3. New IC Chip Producing Process

**Figure 7:**
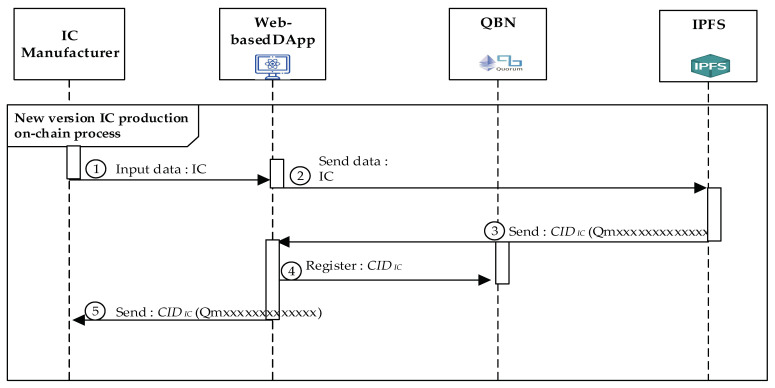
The sequence diagram of producing the new version of IC.

ICM could also produce new versions of ICs without receiving some orders from MM or SM. Figure 7 illustrates the ICM participant in developing new versions of IC chips. The detailed steps of ***New IC Chip Producing Procedure*** are shown below.

***New IC Chip Producing Procedure***

Step 1. ICM registers the data of the produced IC chip with the last version to IPFS through the DApp.
Step 2. After receiving the registering IC data sent from ICM via DApp, IPFS will store them and generate a new and corresponding *CID_IC_* hash value.
Step 3. The DApp will automatically register this corresponding *CID_IC_* value using the deployed DApp smart with the specific contracts we designed into QBN.
Step 4. Finally, DApp will send back the generated *CID_IC_* to ICM. Additionally, the IC product data, stored in IFPS, are also registered with the last version via recording its corresponding new *CID_IC_* value into the QBN performed by the DApp and the related smart contracts.
Step 5. The IPFS will deliver the corresponding *CID_IC_* value back to ICM.

Process transactions in a blockchain-enabled framework based on QBN show how each stakeholder involved in the blockchain quorum network interacts. ICM as an IC manufacturer, MM as a module manufacturer, and SM as a system interact to get the required information. The transactions indicate the blockchain node network authority, SM as a node manager, which will return the public and private keys and corresponding digital identity credentials.

Furthermore, the DApp together with the smart contracts, which are deployed and managed by SM, are performed in order to involve the new information of the IC chip into the new trust chain.

## 5. Experimental Results

**Figure 8:**
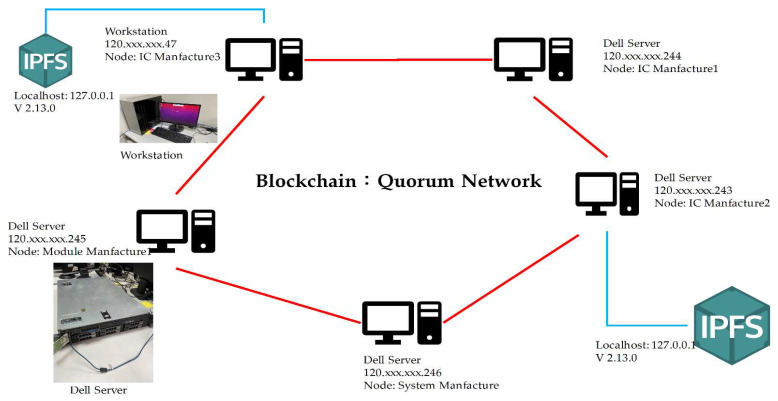
The PowerEdge R710-Dell server is applied to construct the consortium blockchain.

The proposed system is implemented using blockchain and IPFS technologies to be an in-depth framework for this work. The QBN holds a unique hash CID generated by stakeholders for each specific product and issued by IPFS. Solidity is a high-level programming language we use to write all smart contracts in this work. The Solidity language supports inheritance, and libraries can be imported and designed in an Ethereum Virtual Machines (EVM) environment. The resources of experiments for this study are adapted PowerEdge R710-Dell server, shown in Figure 8, to construct a consortium blockchain. Its specifications consist of 2.26 GHz 6 GB Intel^®^ Xeon^®^ 5000 Sequence 570 W 2.26 GHz, E5520, 6 GB, DDR3-SDRAM, 160 GB, and Ubuntu 20.04 x64. Furthermore, one node contains an Intel(R) Core (TM) i7-9700F processor running at 3.00 GHz dual OS Linux Ubuntu 20.04.3 and Windows 10. Remix, Truffle, and Visual Studio as support tools.

Additionally, the six actors are involved in the framework with the secure decentralized trust chain for the developed system. The roles and duties are described below.

SM: This actor manages the design to create a new system. Production of the system requires a license from the system manufacturer. The chain only works when the system manufacturer decides to build a new system. Each part of the new system is assigned a unique identification code.

1. MM: This actor supplies the module material to manufacture the new system. SM will initiate communication with MM by sending the required manufacturing license and ordering information.
2. ICM: It will produce information regarding IC ordered by MM. ICM is responsible for converting raw materials into IC chips. The ICM will provide a unique identification code for each completed IC chip.
3. QBN: Determines the legitimacy of participants/nodes joining the blockchain architecture. Manage constantly increasing groups of records. Collections of transactions are grouped and allocated ledger chain blocks to blocks.
4. IPFS: This decentralized protocol provides secure data storage and a way to generate addressable hashes of uploaded files. The IPFS protocol allows synchronized data distribution. Implementing a distributed hash table (DHT) and a CID-based storage system.
5. DApp: It is a software program that uses smart contracts. Smart contracts could be accessed through DApp, which offers a convenient interface. The DApp that runs on a blockchain network is an example of a cryptocurrency application.

First, each participant, for example, SM, MM, and ICM, will register on QBN, individually. After registration processing, each participant will be assigned a private-public key pair according to Equations (1)–(3) in order to identify his or her unique member on the QBN.

**Figure 9:**
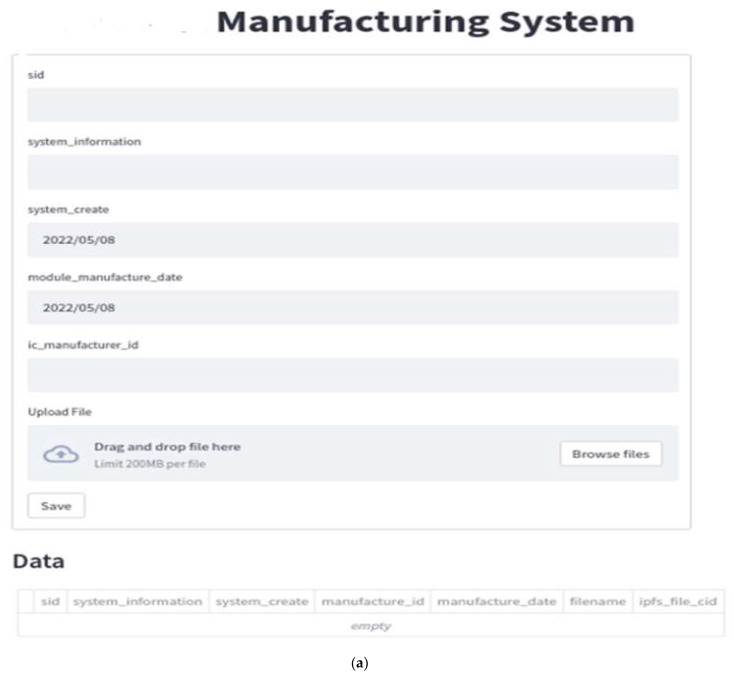

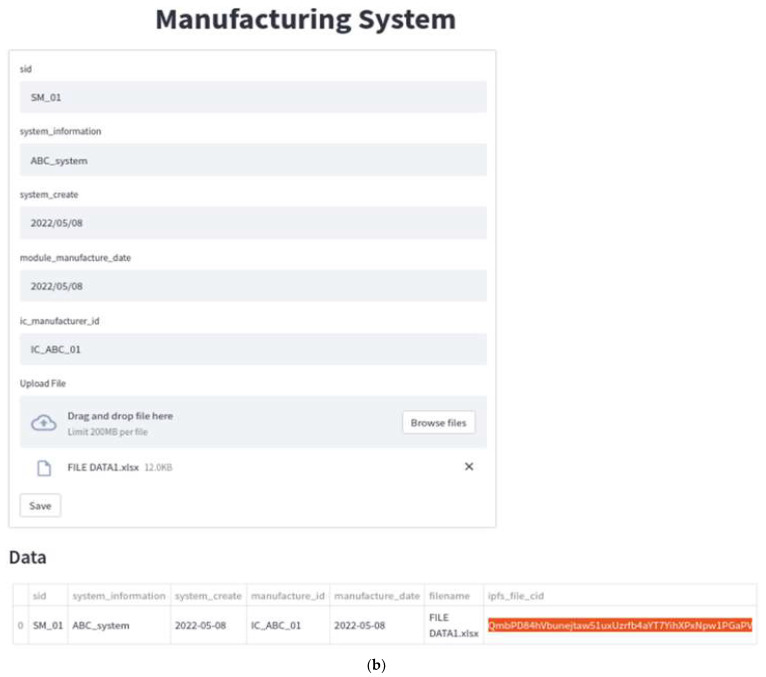
(**a**) DApp of SM (this SM could be called a new MS production); (**b**) DApp of SM with the input data.

After ICM completes IC production, ICM will create an IC chip, either based on the design order agreed with MM or a new version. ICM will also upload the complete IC data and the corresponding files via DApp, as shown in Figure 9. From IPFS, the CID with 46 characters beginning with the character “Qm” is automatically generated. Then, it will be registered on the blockchain by using the deployed smart contract.

After receiving the orders from SM, MM will start to produce the new module which should be fulfilling all the requirements including the requested ICs. MM will then register each generated data and the corresponding CID of this new module into QBN via the implemented smart contract. IPFS will also provide the CID value after the MM uploads the completed module file. MM uses DApp to interact with IPFS and QBN, as shown in Figure 9. Once all the required modules have been completed and accepted by SM with the specified specifications, SM will start producing a new system (called an “MS” system in this paper) or a new version of the existing system.

SM will immediately start recording data and uploading files via DApp after creating the product, as shown in Figure 9. IPFS will generate CID for files containing new system information or upgraded version.

**Figure 10:**
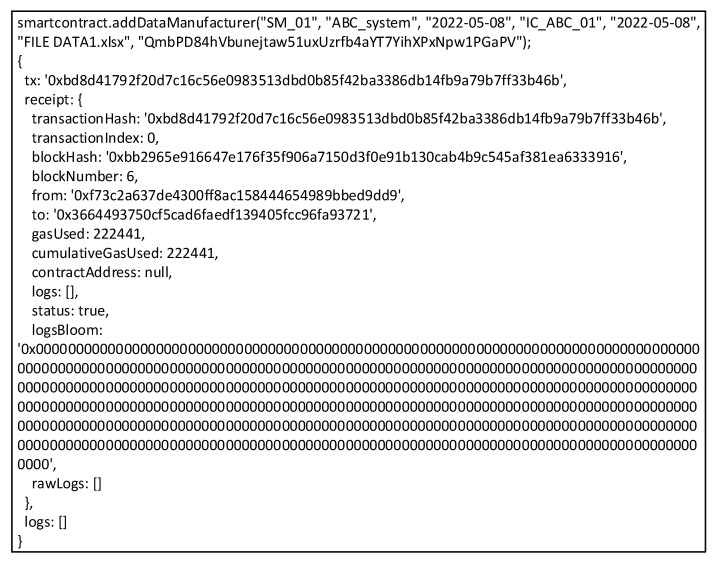
Example of transaction Hash.

QBN executes smart contracts, propagated via Web3 Application Programming Interface (API) to enter data and CID into blockchain with transaction hash result “0xbd8d41792f20d7c16c56e0983513dbd0b85f42ba3386db14fb9a79b7ff33b46b” and with CID number “QmbPD84hVbunejtaw51uxUzrfb4aYT7YihXPxNpw1PgaPV,” as seen in Figure 10. The files would be uploaded to IPFS, which could be in various forms, such as text, images, videos, etc. ICM sends data to MM and uploads to IPFS about built ICs, and MM does the same to support the creation of new systems. SM’s final phase is to generate a new system and upload it to IPFS. The result of the SM file uploading is in the form of master data consisting of several modules incorporated into the new system. The module also consists of several integrated ICs, so that the data stored in IPFS by SM will be in the form of a multilevel hierarchy. All stakeholders could ensure that the new dataset system follows a pre-agreed design.

Public, private, or consortium blockchains could be been categorized according to their real applications and requirements. In general, both public blockchain and consortium blockchain are usually used for public networks. A public blockchain is a permission-less, non-restrictive, distributed ledger system, meaning that anyone with internet access right could join and participate in a blockchain network. The public blockchain maintains the confidence of the entire users’ communities because everyone in the network feels motivated to contribute to the improvement of the public network. However, it suffers from both scalability and time-consuming issues. On the other hand, a consortium blockchain is best suited for organizations that need to implement both private and public systems. In this type, there are multiple central authorities or multiple organizations that provide access to pre-selected nodes for reading, writing, and auditing the blockchain. It maintains its decentralized nature because no single authority governs the control. The cost of production is also relatively lower than the previous public systems. Thus, this section compares the public blockchain represented by the Rinkeby test network [41] with the consortium blockchain [45] represented by Quorum. 

Furthermore, the cost of operating our system is computed with minor state changes, execution moves, or storage space utilization, and we decide to pay miners in Ether. In Ethereum, gas is the measurement for the cost of the execution task. When the user chooses to change the status of such a smart contract, the user must pay for the corresponding state modification gas. Different program operations require different amounts of gas to run. Gas has a fixed operating cost. For example, each contracting process costs 164,175 Wei gas on block 1, and the cost of a contract call on block 2 is 42,431 Wei gas. The charge for each block can be seen in Table 4. On the public blockchain, users can set gas prices for any Wei. However, the higher the gas price, the faster transactions are completed within a block because miners prefer higher-priced transactions. The relationship between both acceptance speed and gas price among the transactions, which is shown in Table 5. The gas price on the consortium blockchain is “0”. EthGasStation is an open-source initiative designed to increase gas price transparency [45]. To pay the smart contract’s procedure cost using Ether, first convert it to US dollars. Navigate the Foreign Exchange (FX) rates website (see https://fx-rate.net/ETH/USD/) to obtain the current exchange.

When experimenting with the scheme, the ether exchange rate was referenced by the Foreign Exchange (FX) website (also referred to on https://fx-rate.net/ETH/USD/ accessed on 14 June 2022); the ether exchange rate reached a high of USD 3810.525 per ether and a low of USD 1,124.493. Furthermore, the median of the highest and lowest, USD 2,726.836, will be the benchmark in calculating costs in our system. Multiplying the gas required to perform the appropriate function by the current value of the ether, we get the price. Utilize Remix to determine the amount of gas required to run our system’s functionalities. The Remix is a web-based integrated development environment (IDE) for developers creating Solidity DApps. The output of the system function execution is shown in Table 6. The evaluation results show that the proposed scheme could work in practice under the much lower costs, compared to the public blockchain, with a total cost o “0.002094” in Ether. According to the highest, the lowest, and the average dollar price as the reference (https://fx-rate.net/ETH/USD/ accessed on 14 June 2022) the most increased initial production cost for the recorded contract is USD 7.97922888. The lowest initial cost is USD 2.354688342, and the average price for each production is USD 5.709994584.

## 6. Discussions and Analyses

The MS with the trust chain of IC-module-system enjoys valuable benefits with the advances in blockchain technology and distributed data processing in terms of flexibility, traceability, and security compared with centralized MS. The proposed framework could manage ubiquitous transactions between manufacturers and communicate with manufacturers to form a trusted chain network that supports more personalized products following ever-changing trends. From a technology point of view, the permissioned blockchain could improve network protection against cyber-attacks with an additional control layer given the drawback of current MS, inviting many parties to join the network, and making direct contact with enterprises. This type of secured blockchain technology also could achieve competitive throughput with low latency. As evident in [38,40,48], the effectiveness of the permissioned blockchain outperforms permissionless blockchain, that is, public Ethereum as a comparison. This study proposes verifiable practices for integrating the permissioned blockchain technology to support tangible insights in applying MS using cases in manufacturing industries. Several instances in manufacturing industries involving ICM, MM, and SM are described by integrating QBN solutions. Additionally, the smart contract and consensus mechanism used in the QBN system significantly impacts the solution’s efficiency. The performance of the proposed method, how costly the implementation, and the energy consumption would be specific considerations for the enterprises to switch to using these technologies.

Due to the complexity of transactions, product traceability, and product counterfeiting, the manufacturing industry faces many challenges. This problem can be solved by using blockchain technology to manage transaction security, and IPFS stores large amounts of distributed data. Transactions in the manufacturing industry are very complicated because of the information, such as product data, payment transactions, and product ownership. A secure chain of trust is needed in the production process to ensure product authenticity and traceability, QBN development with IPFS can be realized efficiently to handle the complexities of MS in the real world. Thus, the application in designing the proposed system framework could answer the above challenges. A secure trust chain built hierarchically from the initial phase of production to the final phase could be a solution to the problems faced in the manufacturing industry.

These three cases, Case 1, 2 and 3, are discussed and analyzed below.

Case 1: IC-module-system data in the trust chain.

**Figure 11:**
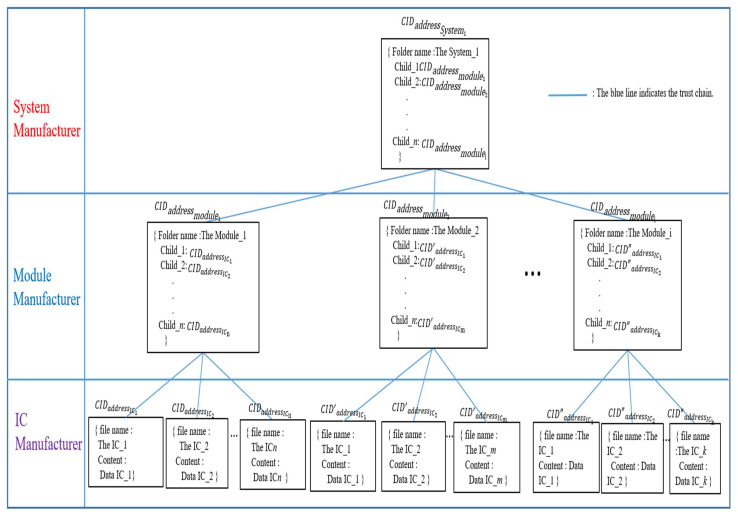
Merkle DAG in HCIDM performed in web-based DApp is activated and organized the trust chain in our framework on when any version data of IC, module or system is recorded.

Merkle DAG [55] is utilized as a data object model for HCIDM, which is analogous to a Merkle tree proposed by Ralph C. Merkle [54]. A structure comprising two properties, Data, and Links, constitutes an IPFS object. Each Link structure includes the Name, Hash, and Size characteristics. Thus, HCIDM could assemble things and construct a directed acyclic graph by utilizing this object structure. Figure 11 depicts how HCIDM employed Merkle DAG to arrange the construction of a file or file directory in a web-based DApp. Figure 11 describes the HCIDM used in the proposed scheme, where the structure of HCIDs consists of three layers, the system manufacturer layer, the module manufacturer layer, and the IC manufacturer layer. In the IC manufacturer layer, it consists of leaf nodes where each leaf node contains a file name and IC data. Each leaf node has a CID hash value, for example, $CID_{address_{IC_{x}}}\in$$\left\{CID_{address_{IC_{1}}},CID_{address_{IC_{2}}},\ldots ,CID_{address_{IC_{n}}}\right\}$, $CI{D^{\prime }}_{address_{IC_{x}}}\in$$\left\{CI{D^{\prime }}_{address_{IC_{1}}},CI{D^{\prime }}_{address_{IC_{2}}},\ldots ,CI{D^{\prime }}_{address_{IC_{n}}}\right\}$ or $CI{D^{\prime \prime }}_{address_{IC_{x}}}\in$$\left\{CI{D^{\prime \prime }}_{address_{IC_{1}}},CI{D^{\prime \prime }}_{address_{IC_{2}}},\ldots ,CI{D^{\prime \prime }}_{address_{IC_{n}}}\right\}$. Each leaf node has a different path to its parent node, the node module. 

The module manufacturer layer consists of several module nodes, where each module node contains the module folder file name and the child’s CID hash value. Each module node has a CID hash value at the same layer, e.g., $CID_{address_{{\mathrm{module}}_{x}}}\in$$\left\{CID_{{\mathrm{address}}_{{\mathrm{module}}_{1}}},CID_{address_{{\mathrm{module}}_{2}}},\ldots ,CID_{address_{{\mathrm{module}}_{n}}}\right\}$. 

At the system manufacturer layer, the customized product, system_1, is the top parent node in the Merkle DAG tree. The top node system_1 contains the folder name and all CID hash values of the modules and ICs via his children links to each module node. The result forms HCIDs of interrelated directories and subdirectories, which are used to create a system-level trust chain.

Case 2: Update the IC-module-system data in the trust chain.

**Figure 12:**
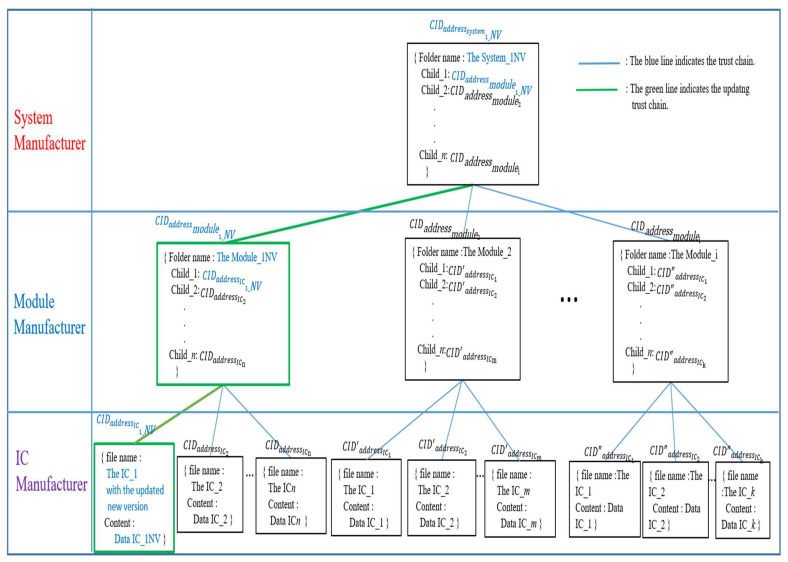
Merkle DAG in HCIDM performed in web-based DApp is activated and re-organized the trust chain in our framework when any version of IC, module, or system is updated.

How to update any version data of IC, module, or system is an additional essential issue that HCIDM has to address. Each IPFS file has an associated hash address. When the IPFS stored file is modified, the hash address will be changed, too. Whenever any new version data of IC, module, or system is added to an IPFS network, it will be assigned a new CID which is a unique hash value of the new version data of IC, module, or system. In traditional IPFS networks, it is identified and referenced in this way. Recalculating the hash when retrieving the file will verify its integrity. Modified files will fail the verification. When a file is legally modified, IPFS takes care of versioning the file. It indicates that the updated version of the file is stored with the new CID, and the previous version of the old file could be kept and retrieved via the previous CID. Figure 12 describes how to update the information for the updated IC version by using Merkle DAG employed by HCIDM. The first step is to create a Mutable File System (MFS) directory [43]. In Case 2, if anyone wants to add or update any version of IV, module, or system in this HCID tree, the hash values of the leaf nodes and subdirectory nodes regarding the related path will be changed, too. In this Case 2, the ICM makes IC_1 with a new version. Then the IC_1 with the new version will get a new CID ($CID_{address_{IC_{1\_\mathrm{NV}}}}$). The module node in Figure 12, which is the parent of the IC node with the new IC_1 version, will update the link directory by updating a new CID value retrieved by IPFS implemented in this framework. After updating all the related files whose links are belonging to updating the Merkle DAG path, for example, $\langle CID_{address_{IC_{1\_\mathrm{NV}}}}↔CID_{address_{{\mathrm{module}}_{1\_\mathrm{NV}}}}↔CID_{address_{system_{1\_\mathrm{NV}}}}\rangle$, the parent module will get a new CID hash value $CID_{address_{{\mathrm{module}}_{1\_\mathrm{NV}}}}$. The system_1 node in the system build layer will also update the directory by updating the CID for the updated module $CID_{address_{{\mathrm{module}}_{1\_\mathrm{NV}}}}$, then the system_1 node will get the new hash value $CID_{address_{system_{1\_\mathrm{NV}}}}$. With this procedure, the trust chain could be updated, via Merkle DAG employed by HCIDM, to be the new latest trust chain according to Definition 1 or Definition 2.

Case 3: Delete the IC-module-system data on the chain of trust.

**Figure 13:**
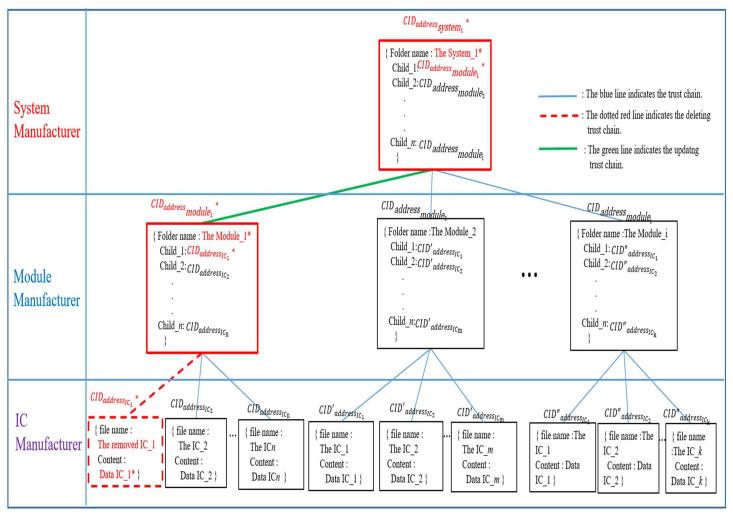
Merkle DAG in HCIDM performed in web-based DApp is activated and re-organized the trust chain in our framework on when IC_1 data is deleted, where the symbol “*” is used to indicate the version of CID address (or said hash value) has been undated.

The HCIDM preserves the history of computing devices by keeping each stored file version and building a solid network for data mirroring. Each participant, ICM, MM, or SM uses the web-based DApp, based on HCIDM, to automatically store, update and manage the version data of their product. Figure 13 describes how a version data file is deleted in the ICM leaf node. ICM will carry out the garbage collection process both manually and automatically. In this case, the file to be deleted has a CID hash value ($CID_{address_{IC_{1}}}{}^{*}$). ICM will delete the version data file regarding an old IC chip in order to revoke it. ICM will then publish the revoked information to the MM who is his parent node. Next, MM will update the file in this IPFS in order to get a new CID hash value $CID_{address_{{\mathrm{module}}_{1}}}{}^{*}$. After getting updated information from the associated child module(s), SM will also update the contents in his folder shown in Figure 13 and get the new CID hash value $CID_{address_{{\mathrm{system}}_{1}}}{}^{*}$. With this procedure, the trust chain could be updated to be the new latest trust chain according to Definition 1 or Definition 2. By carrying out this procedure, the security for the trust chain could also remain secure and solid. 

## 7. Conclusions

The proposed framework with a comprehensive identification mechanism is implemented in this paper. It integrates the three latest advances in computer engineering, including the permissioned blockchain, DApp, and distributed database mechanism, in order to implement the comprehensive identification mechanism with the trust chain of IC-module-system for MS toward secure Industry 4.0. The results show that it is flexible and traceable, enabling current MS with a comprehensive identification mechanism for achieving a more secure and reliable MS. This study emphasized using system architecture and proof-of-concept algorithms by integrating several components, including the permissioned blockchain network, smart contracts, and a consensus mechanism. In addition, to validate the proposed architecture, simulations were carried out using the QBN, DApps, and IPFS database. The results also revealed significant contributions. Firstly, each object in the current MS connected to a centralized system makes the system hard to scale, trace, and monitor. A decentralized system provides a more scalable network achieving a more flexible implementation while maintaining security issues. Secondly, the proposed method works at a much lower price compared to the public blockchain. Moreover, the highest initial production cost for the recorded contract is USD 7.97922888, the lowest initial price is USD 2.354688342, and the average cost for each production is USD 5.709994584, where they were calculated on 14 June 2022, via accessing the referred website on https://fx-rate.net/ETH/USD/. Thus, its implementation to face the challenges of current MS is feasible. Finally, blockchain technology, decentralized database, and DApp offer the practical concept to improve the current MS concept. However, there are several limitations in this work such as market transparency and verifiability. Based on insights obtained from this analysis, the trust chain based on HCIDM can be applied to any MS system, for example, this trust chain could be used to prevent the counterfeit modules and ICs employed in the monitoring system of semiconductor factory environment. Finally, this research is developed an innovation trust chain mechanism which could be provided the system-level security for any MS toward Industrial 4.0 in order to meet the requirements of both manufacturing innovation and product innovation in Sustainable Development Goals (SDGs). In future work, this study will enhance current efforts on extending the proposed system to achieve a secure end-to-end transparent and verifiable supply chain.

## Figures and Tables

**Table 1:** Comparison with various manufacturing systems.

Authors	Year	Objective	Technologies
Zhang, C. et al. [12]	2020	Their permissioned blockchain could achieve better throughput and lower latency than the permissionless blockchain, making it more resource-efficient and appropriate for MBCoT.	Integrates IIoT with the permissioned blockchain.
Zhong, R.Y et al. [18]	2017	This article presented a framework of Industry 4.0-based IMS, in which research topics are categorized into smart design, smart machines, smart monitoring, smart control, and smart scheduling.	The IoT, CPSs, cloud computing, big data analytics, and other information and communication technologies (ICTs).
Ma, J. et al. [24]	2020	Manufacturers could use the system to store relevant information on product sales in blockchain which is accessible to everyone.	Based on Ethereum blockchain.
Xiao, L et al. [25]	2020	The secure, intelligent contract protocol of Intellectual Property circuit protection under the blockchain environment further enhances security and reliability.	The blockchain environment.
Heo, G et al. [26]	2021	The proposed SBBC system includes off-chain and on-chain modules to establish secure and reliable digital content trading.	The secret block-based blockchain (SBBC).

**Table 2:** The code structure of smart contract.

The System Code Structure	The Types of Roles
Type system struct{ S_ID string system_inform string system_Crea_Datetime time.Time Module_manufac_ID string Module_manufac_Datetime DateTime IC_Manufac_ID string IC_ Manufac _Datetime DateTime MS_Sign string MM_Sign string ICM_Sign string } Type Module struct{ M_ID string Module_manufac_Datetime DateTime IC_Manufac_ID string IC_ Manufac _Datetime DateTime MM_Sign string ICM_Sign string } Type IC struct{ IC_ID string IC_inform string IC_Manufac_ID string IC_ Manufac _Datetime DateTime ICM_Sign string }	Type Roles string const{ System Manufac Module Manufac IC Manufac } Type Node_table struct{ Node_name string Role string public-key string IP address Enode_ID string }

**Table 3:** Notations.

$ID_{r}$	The Identity of a Manufacturer. For example, System Manufacture, Module Manufacture, and IC Manufacture.
SM	The System Manufacturer.
MM	The Module Manufacturer.
ICM	Ihe IC manufacturer.
$Kpri_{\;r}\;$	The private key for participant *r.*
$Kpub_{r}$	The public key for participant *r*.
$Kaddr_{r}$	The address key for participant *r*.
*G*	A generating point based on Elliptic Curve E.
*S_IDi*	S_ID is the system’s *i*th identifier (blockchain address).
*M_IDi*	M_ID is the module’s *i*th identifier (blockchain address).
*IC_IDi*	IC_ID is the system’s *i*th identifier (blockchain address).
*E*	The Elliptic Curve is defined as a finite group.
*Ti*	It is the *i*th timestamped value.
Δ *T*	The validity threshold of the time stamp.
*M_SM_*	The nessage sent by the system manufacturer.
*M_MM_*	The message sent by the module manufacturer.
*M_ICM_*	The message sent by the IC manufacturer.
*(R_ri_,S_ri_)*	The Elliptic Curve signature value for the role *r*.
*(A_ri_,B_ri_)*	The value of the ECDSA signature for role *r*.
*E_pukx_ (M)/E_prkx_ (M)*	Encrypt or Decrypt Algorithm the message *M* by using the public key or private key party of *role r*, separately
*s*	The indicator of design system.
*m*	The indicator of design module.
*ic*	The indicator of design IC.

**Table 4:** The comparison of transaction costs between Truffle and QBN operation using 12 blocks.

Transaction	Gas Used	Gas Price		Transaction Cost		Block No.
		Public Blockchain [41]	Consortium Blockchain [45]	Public Blockchain [41]	Consortium Blockchain [45]	
No. transaction	0	0.000042 Eth	0 Eth	0	0	0
Contract Creation	164,175	0.000042 Eth	0 Eth	6.89535	0	1
Contract Call	42,431	0.000042 Eth	0 Eth	1.782102	0	2
Contract Creation	96,189	0.000042 Eth	0 Eth	4.039938	0	3
Contract Call	27,341	0.000042 Eth	0 Eth	1.148322	0	4
Contract Creation	222,254	0.000042 Eth	0 Eth	9.334668	0	5
Contract Call	27,341	0.000042 Eth	0 Eth	1.148322	0	6
Contract Call	84,015	0.000042 Eth	0 Eth	3.52863	0	7
Contract Call	26,415	0.000042 Eth	0 Eth	1.10943	0	8
Contract Call	34,815	0.000042 Eth	0 Eth	1.46223	0	9
Contract Call	38,415	0.000042 Eth	0 Eth	1.61343	0	10
Contract Call	34,815	0.000042 Eth	0 Eth	1.46223	0	11

**Table 5:** The relationship of both acceptance speed and gas price among the transactions.

Transaction Speed	Gas Price
Very likely in <15 s, high	Max fee:(0.000042 ETH)
Likely in <30 s, medium	Max fee:(0.0000315 ETH)
Maybe in 30 s, low	Max fee:(0.00002961 ETH)

**Table 6:** The gas cost in executing functions in the proposed framework.

	Costs	Gas Used (Units)	Gas Price: (Gwei)	Gas Fee (Gwei)	Gas Fee (Ether)	Total Cost (US Dollar)
The Description of Functions						
Deploy_the_contract		608,245	2.500000866	1,520,613.027	0.001521	1.85
Send_account		43,836	2.500000866	109,590.038	0.00011	0.13
Send_IPFS_cid		90,028	2.500000866	225,070.078	0.000225	0.27
Received_address		68,365	2.500000866	170,912.5592	0.000171	0.21
Send_address		26,760	2.500000866	66,900.02317	0.000067	0.08
		Total: 837,234		Total: 2,093,085.725	Total: 0.002094	Total: 2.54

## Data Availability

Not applicable.
